# Assessment of Surrogate End Point Trends in Clinical Trials to Approve Oncology Drugs From 2001 to 2020 in Japan

**DOI:** 10.1001/jamanetworkopen.2023.8875

**Published:** 2023-04-28

**Authors:** Hideki Maeda, Riko Shingai, Kentaro Takeda, Asuka Hara, Yuna Murai, Momoka Ofuchi

**Affiliations:** 1 Department of Regulatory Science, Faculty of Pharmacy, Meiji Pharmaceutical University, Noshio, Kiyose-city, Tokyo, Japan; 2 Data Science, Astellas Pharma Global Development, Inc, Northbrook, Illinois

## Abstract

**Importance:**

A surrogate end point (SEP) is an end point used in clinical trials as an alternative for measuring the true clinical benefit. The use of SEPs in trials shortens their duration.

**Objectives:**

To investigate the use of SEPs in clinical trials to support the approval of anticancer drugs and to determine whether confirmatory studies that use overall survival (OS) as an end point are being conducted in Japan.

**Design, Setting, and Participants:**

In this cross-sectional study, drug approvals and background information were obtained from publicly available information, such as the Pharmaceuticals and Medical Devices Agency website, for anticancer drugs approved in Japan from January 2001 to December 2020. Data analysis was performed from September 2021 to March 2022.

**Main Outcomes and Measures:**

Characteristics of approved oncology drugs in Japan, end points for pivotal clinical trials, and outcomes of confirmatory trials using OS as an end point following drug approval.

**Results:**

There were 299 anticancer drugs approved in Japan during the study period. Of these, 142 (47.5%) were molecular-targeted drugs, the most common of which targeted non–small cell lung cancer. There were 111 (37.1%) anticancer drugs with orphan designation. From 2001 to 2005, OS was used as an end point in 1 approval (3.6%); however, from 2006 to 2020, OS was used in 86 approvals (31.7%). Of the 212 anticancer drugs approved on the basis of SEPs, confirmatory studies with OS as the end point were conducted for only 37 approvals (17.5%); for the remaining 175 approvals, studies are under way for 35 approvals (16.5%), were waivered for 75 approvals (35.4%), and were not conducted for 65 approvals (30.7%). Furthermore, in 20 drug approvals (9.4%), the conducted confirmatory studies were not effective in determining the OS, but the drugs were approved following re-examination.

**Conclusions and Relevance:**

The findings of this study suggest that starting from 2005, the use of OS as an end point has increased in studies supporting the approval of anticancer drugs in Japan. However, even after 2005, approximately two-thirds of these approvals were SEP based. Postmarketing surveillance studies of the true end points are necessary to validate the use of SEPs.

## Key Points

**Question:**

How often are surrogate end points used to support the approval of anticancer drugs, and are confirmatory studies that use overall survival as an end point conducted in Japan?

**Findings:**

In this cross-sectional study of 299 anticancer drugs, the number of drugs for which overall survival was used to support approval increased from 1 in 2001 to 2005 to 86 in 2006 to 2020; however, 212 approvals were based on surrogate end points. Postmarketing confirmatory studies using overall survival as an end point were not conducted for 175 approvals.

**Meaning:**

These findings suggest that the Japanese regulatory agencies need to evaluate approval through surrogate end points and ensure the implementation of postmarketing commitment studies.

## Teaser

This cross-sectional study investigates the use of surrogate end points in Japanese clinical trials to support the approval of anticancer drugs and examines whether confirmatory studies that use overall survival as an end point are being conducted.

## Introduction

Overall survival (OS) is a hard end point for oncology-related clinical trials and is considered the criterion standard.^1,2,3^ Apart from the Pharmaceuticals and Medical Devices Agency in Japan, the US Food and Drug Administration (FDA), the European Medicines Agency, and other regulatory agencies in high-income countries have continuously applied this standard for approval of anticancer drugs (new drug OS is required as an end point of a pivotal clinical study included in the clinical data package at the time of administration for new drug applications [NDAs]).^4,5,6,7,8^

In particular, the guidelines for the clinical evaluation of anticancer drugs issued in February 1991 represent the first set of regulations for the clinical development of anticancer drugs in Japan.^9^ In line with these guidelines, regulatory agencies in the past would grant approval for anticancer drugs on the basis of the results of phase 2 studies that mainly relied on response rate as a surrogate end point (SEP). However, the guidelines for the approval of anticancer drugs were revised,^10^ and the updated guidelines were released in April 2006. The revised guidelines clearly state that for certain major types of cancer, including non–small cell lung cancer (NSCLC), gastric cancer (GC), colorectal cancer (CRC), and breast cancer (BC), results from phase 3 studies relying on end points demonstrating life-prolonging effects, such as OS, must be submitted at the time of application. However, it is likely that many approvals based on SEPs have been actually granted in Japan after 2006. The problem with conducting clinical trials that use OS as the end point is that the duration of the trial needs to be longer than those for trials using alternative end points, leading to a process that may span several years.^11^ Including SEPs can reduce the time required for oncology trials and, thus, lead to the faster practical application of anticancer drugs. However, it is paramount to strictly consider the effectiveness of the drug and the acceptability of the SEP,^12^ with the ultimate aim of delivering novel therapies to patients in a quick manner.

The FDA provides a list of SEPs, including 17 SEPs for adult cancer and 6 SEPs for pediatric cancer (as of August 31, 2022).^13^ Furthermore, when using SEPs, it is normal to perform validation using tools such as the Prentice Criteria; however, in carcinomas such as BC,^14^ CRC,^15^ GC,^16^ lung cancer,^17,18^ renal cancer,^19^ brain tumor,^20^ leukemia,^21^ or those treated with immune checkpoint inhibitors,^22^ OS and SEP correlation studies are being conducted, and validation is being considered. Maeda et al^23^ have also examined the validation of SEP in prostate cancer and recently presented the results. In 2014, Maeda et al^23,24^ conducted a research study on SEP usage in Japan. However, to the best of our knowledge, no relevant studies have been conducted after the recent policies (ie, Sakigake designation and conditional early approval system) were implemented to promote the development of innovative anticancer drugs, such as immune checkpoint inhibitors and molecular-targeted drugs.

Japan has a unique re-examination system. This system includes a secondary approval step in which the efficacy and safety of newly approved drugs are reviewed in the postmarketing setting. The main purpose of this re-examination step is to confirm drug safety, but if there were any concerns regarding drug efficacy in the initial approval, these are also considered. Currently, there is a lack of research on postmarketing clinical studies and the approval requirements for anticancer drugs in Japan. Therefore, in this study, we have comprehensively investigated the end points of pivotal clinical trials at the time of approval of anticancer drugs in Japan and examined whether postmarketing confirmatory studies that use OS as an end point have been conducted.

## Methods

This cross-sectional study did not include individual patient records. Instead, it used publicly available data. Therefore, institutional review board approval and patient informed consent were not necessary, in accordance with 45 CFR §46. This study was prepared in accordance with the Strengthening the Reporting of Observational Studies in Epidemiology (STROBE) reporting guideline^25^ for cross-sectional studies. Supplemental information and details regarding the methods used in this study are presented in eTable 1 in Supplement 1.

### Selection of Drugs and Databases

In this study, we have investigated the oncology drugs approved in Japan from January 2001 to December 2020. We included initial NDAs (iNDAs) as new molecular entities, as well as additional indication supplemental NDAs (sNDAs).

### Data Collection

Data were collected from publicly available databases through the Pharmaceuticals and Medical Devices Agency website.^26^ We followed the methods of data collection and extraction outlined in our previous study.^27^

### Statistical Analysis

Data analysis was performed from September 2021 to March 2022. Descriptive statistics were used to characterize the samples of the new drugs and their indications. The 2-sided χ^2^ test was used to evaluate the changes in end points for pivotal clinical trial–supported approvals, with significance set at *P* < .05. A logistic regression model was used to examine the association between the SEPs and background factors. All analyses were conducted using SAS statistical software version 9.4 (SAS Institute) and JMP Pro statistical software version15 (JMP Statistical Discovery).

## Results

### Regulatory and Clinical Characteristics of Approved Oncology Drugs in Japan

From January 2001 to December 2020, 299 anticancer drugs were approved in Japan; of these, 122 were iNDAs, and 177 were sNDAs. The annual changes in the number of approved anticancer drugs over the past 20 years are shown in the Figure. The number of anticancer drugs approved in Japan has been increasing year by year, and in 2020, there were 36 approvals, which is the largest number to date.

**Figure.**
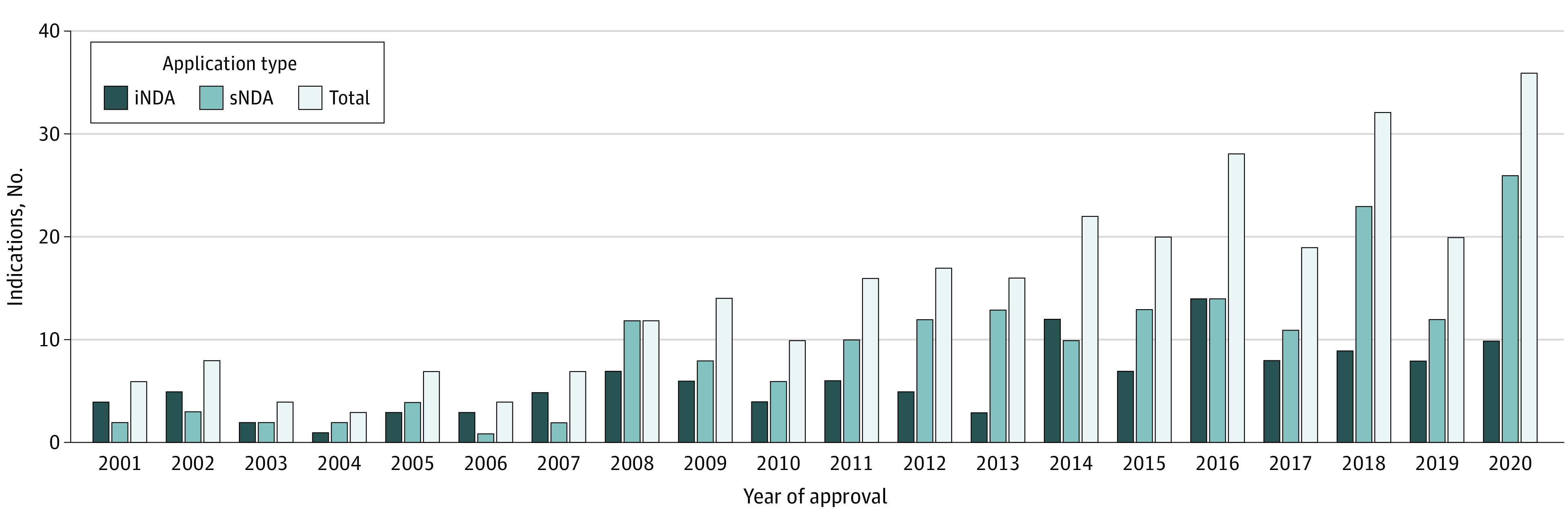
Number of Approvals for Oncology Drugs From 2001 to 2020 in Japan iNDA indicates initial new drug application; sNDA, supplemental new drug application.

The clinical characteristics and regulatory backgrounds for the 299 approved drugs are shown in Table 1. Molecularly targeted drugs, in terms of mode of action, were the most common (142 approvals [47.5%]). Indications were major cancers (NSCLC, BC, CRC, and GC) with 87 approvals (29.1%). Solid cancer was also common (208 approvals [69.6%]). The most common types of cancers overall were NSCLC (34 approvals [11.4%]), BC (27 approvals [9.0%]), and CRC (17 approvals [5.7%]). Furthermore, 111 approvals (37.1%) were given for orphan drug designations, and 30 approvals (10.0%) were public knowledge–based applications that did not have clinical trials for approval application. For the development style, 127 approvals (42.5%) were conducted using a global multiregional strategy joint international study, followed by those that used a bridging strategy using data from overseas phase 3 studies (109 approvals [36.5%]). In addition, 246 approvals (82.3%) were applied using overseas data. Furthermore, 218 (72.9%) of the approved drugs had already been approved by the FDA at the time of approval in Japan, and 170 (56.9%) of the approved drugs were administered using injections.

**Table 1.** Characteristics of the Approved Oncology Drugs From 2001 to 2020 in Japan

Characteristic	Drugs, No. (%) (N = 299)
NDA	
iNDA	122 (40.8)
sNDA	177 (59.2)
Mode of action	
Cytotoxic drug	95 (31.8)
Molecularly targeted drug	142 (47.5)
Immunotherapy	37 (12.4)
Hormonal drug	14 (4.7)
Antibody drug conjugate	3 (1.0)
Others	8 (2.7)
Companion diagnostics	
Yes	82 (27.4)
No	217 (72.6)
Tumor type	
Major cancer	87 (29.1)
Non–small cell lung cancer	34 (11.4)
Breast cancer	27 (9.0)
Colorectal cancer	17 (5.7)
Gastric cancer	9 (3.0)
Not major cancer	212 (70.9)
Multiple myeloma	15 (5.0)
Prostate cancer	13 (4.3)
Melanoma	13 (4.3)
Renal cell carcinoma	11 (3.7)
Non-Hodgkin lymphoma	10 (3.3)
Chronic lymphocytic leukemia	9 (3.0)
Ovarian cancer	8 (2.7)
Cervical cancer	8 (2.7)
Hepatocellular carcinoma	8 (2.7)
Acute lymphatic leukemia	8 (2.7)
Other solid tumor	59 (19.7)
Other hematological cancer	50 (16.7)
Solid cancer or hematologic cancer	
Solid cancer	208 (69.6)
Hematologic cancer	91 (30.4)
Limitation of indication	
Resistant or second line or higher	98 (32.8)
Nothing or first line	201 (67.2)
Special designation by Pharmaceuticals and Medical Devices Agency	
Orphan drug designation	111 (37.1)
Normal application	104 (34.8)
Priority review	53 (17.7)
Public knowledge–based application	30 (10.0)
Expedited review	15 (5.0)
Sakigake designation	6 (2.0)
Pediatric disease designation	6 (2.0)
Conditional early approval system	3 (1.0)
Development style	
Global multiregional strategy	127 (42.5)
Bridging strategy	109 (36.5)
Domestic strategy	30 (10.0)
Other style (eg, public knowledge–based application)	33 (11.0)
Using foreign clinical data for NDA package in Japan	
Yes	246 (82.3)
No	53 (17.7)
Special committee on unapproved drug in Japan	
Yes	58 (19.4)
No	241 (80.6)
All case investigation after approval in Japan	
Yes	115 (38.5)
No	229 (76.6)
Postmarketing clinical study requirement in Japan	
Yes	18 (6.0)
No	281 (94.0)
Special designation by FDA	
Priority review and/or orphan designation	81 (27.1)
Fast track designation	32 (10.7)
Accelerated approval	37 (12.4)
Breakthrough therapy designation	11 (3.7)
FDA approval when Japan approval	
Yes	218 (72.9)
No	81 (27.1)
Patients in pivotal clinical study, No.	
<100	63 (21.1)
≥100	236 (78.9)
Domestic vs foreign company	
Domestic company	108 (36.1)
Foreign company	191 (63.9)
Origin of product	
Japan	59 (19.7)
Foreign country	240 (80.3)
Biological drug	
Yes	88 (29.4)
No	211 (70.6)
Formulation	
Oral	121 (40.5)
Injection	170 (56.9)
Others	8 (2.7)

Abbreviations: FDA, US Food and Drug Administration; iNDA, initial new drug application; NDA, new drug application; sNDA, supplemental new drug application.

### Characteristics of End Points That Support Approval

Changes in the pivotal study end points at the time of application for approval for the 299 approvals surveyed every 5 years from 2001 are shown in Table 2. A significant change was observed for each end point and transition every 5 years. From 2001 to 2005, 26 approvals (92.9%) had a response rate, whereas only 1 approval (3.6%) had OS as the end point of the pivotal study, but from 2006 to 2010, 86 approvals (31.7%) had OS as their end point and 19 (42.2%) had response rate as their end point. OS was the end point for 26 approvals (28.6%) from 2011 to 2015 and for 46 approvals (34.1%) from 2016 to 2020. SEPs were used for 89 of 135 approvals (65.9%). The response rate was the end point of 30 approvals (33.0%) from 2011 to 2015 and 36 approvals (26.7%) from 2016 to 2020. Progression-free survival as an end point has been increasing since 2006, reaching 28.9% (39 approvals) from 2016 to 2020.

**Table 2.** Changes of End Points for Pivotal Clinical Trials–Supported Approvals^a^

End point	Trials, No. (%)				
	2001-2005 (n = 28)	2006-2010 (n = 45)	2011-2015 (n = 91)	2016-2020 (n = 135)	Total (N = 299)
Overall survival	1 (3.6)	14 (31.1)	26 (28.6)	46 (34.1)	87 (29.1)
Response rate	26 (92.9)	19 (42.2)	30 (33.0)	36 (26.7)	111 (37.1)
Progression-free survival	0	3 (6.7)	26 (28.6)	39 (28.9)	68 (22.7)
Time to progression	0	5 (11.1)	3 (3.3)	0	8 (2.7)
Disease-free survival	0	3 (6.7)	3 (3.3)	6 (4.4)	12 (4.0)
Other end points	1 (3.6)	1 (2.2)	3 (3.3)	8 (5.9)	13 (4.3)

^a^ *P* < .001 for all comparisons.

Furthermore, to examine the characteristics of approvals using SEPs, the contributions of each factor were examined using logistic regression with the presence or absence of SEP as the objective variable and all the background factors in Table 1 as explanatory variables. The associations between the SEPs and each background factor are shown in eTable 2 in Supplement 1. By selecting the model with the smallest Akaike Information Criterion value among all possible models, it was evident that companion diagnosis and orphan drug designation contributed to accelerating using SEP, whereas the sponsor (domestic company), biologics, tumor type (solid cancer), and priority review contributed to accelerating approvals using OS (Table 3).

**Table 3.** Multivariate Logistic Regression Analysis Based on a Model With the Smallest Akaike Information Criterion Among All Possible Models Relating to the Acceptance of Surrogate End Points and Background Factors

Parameter	Estimate (SE) [95% CI]	*P* value
Intercept	0.930 (0.060) [0.812 to 1.048]	<.001
Companion diagnosis	0.145 (0.052) [0.042 to 0.248]	.006
Orphan drug designation	0.096 (0.053) [−0.008 to 0.200]	.07
Sponsor (domestic or foreign)	−0.080 (0.047) [−0.172 to 0.011]	.09
Biologics	−0.185 (0.051) [−0.285 to −0.084]	<.001
Tumor type (solid cancer or hematologic cancer)	−0.217 (0.055) [−0.326 to −0.109]	<.001
Priority review	−0.333 (0.063) [−0.457 to −0.208]	<.001

### Follow-up of Oncology Drug Approvals Based on SEP

The end points of this pivotal study that assessed the data from 299 approved anticancer drugs over the past 20 years are shown in Table 4 and the eFigure in Supplement 1. Of these drugs, 87 were approved using OS as the end point, and 212 were approved using SEPs.

**Table 4.** Confirmatory Studies Using Overall Survival After Approval

Characteristic	Studies, No. (%) (N = 299)
Approved by overall survival	87 (29.1)
Approved by surrogate end point	212 (70.9)
Completed confirmatory study with overall survival	37 (17.5)
Positive result	15 (7.1)
Negative result but approved by re-examination from other evidence	20 (9.4)
Negative result	2 (0.9)
Ongoing confirmatory study with overall survival	35 (16.5)
Waiver of confirmatory study with overall survival	75 (35.4)
Not yet conducted confirmatory study with overall survival	65 (30.7)

Of the 212 anticancer drugs approved on the basis of SEPs, 37 (17.5%) underwent confirmatory studies with OS as the end point after approval; in 20 drug approvals (9.4%), the conducted confirmatory studies were not effective in determining the OS, but the drugs were approved following re-examination. For the remaining 175 approvals, as of December 2020, 35 confirmatory studies (16.5%) were still ongoing, 75 drugs (35.4%) were exempt from confirmatory studies because the drug was approved either through bridging studies that had available overseas OS data or public domain applications at the time of approval, and 65 drugs (30.7%) did not undergo confirmatory studies and were approved through re-examination on the basis of other evidence of safety or efficacy. More than one-half of the anticancer drugs (22 approvals [59.5%]) that had undergone confirmatory studies were approved in re-examination.

## Discussion

In this cross-sectional study, we have summarized the end points of pivotal studies used for the approval of anticancer drugs in Japan over 20 years. To the best of our knowledge, this is the first long-term and comprehensive study of end points to support Japanese drug approvals. This study also clarified trends in the regulatory and clinical characteristics of anticancer drugs approved in Japan over the study period.

In Japan, from 2001 to 2020, the following 3 major changes have occurred: (1) revision of anticancer drug guidelines^10,28^; (2) emergence of a drug lag problem (ie, a delay in drug approval compared with other countries) and countermeasures^29,30^; and (3) the establishment of guidelines for international clinical trials.^31,32^ These 3 events have substantially changed the methods used for the clinical development of anticancer drugs and the design of clinical trials, and they have also had a substantial impact on end points. In addition, to eliminate drug lag and obtain early approval of drugs that have already been approved overseas, public knowledge filing was established.^33^ Similarly, a bridging strategy that extrapolates overseas phase 3 study results was used to bypass the need for conducting phase 3 studies in Japan to help eliminate drug lag.^34^ Subsequently, guidelines for international joint trials were enacted, and Japan actively participated in global joint trials and in simultaneous global development to help eliminate drug lag.^29^ The results of this study show that the changes over time in the development strategy have coincided with the aforementioned changes.

Furthermore, according to the results of our study, before 2005, most of the approvals were based on the response rate, and after 2006, the number of approvals that were based on the OS increased, showing a significant change. According to these results, the revision of the guidelines is considered to have had a strong impact (Table 2). Using the US as a reference, of the 225 pivotal studies of anticancer drugs approved by the FDA in 2020, 65 (28.9%) had an OS end point, and 158 (70.2%) had an SEP.^35^ In addition, it has been reported that approximately one-third of the anticancer drugs approved by the FDA from 2006 to 2018 were approved on the basis of their response rate.^36^ Our most recent results for 2016 to 2020 show that OS approval was 34.1% and SEP approval was 65.9%, of which the response rate approval was 26.7%, similar to the results from the US (Table 2). The results are reasonable considering that 82.3% (246 of 299 approvals) of pivotal overseas studies were used in Japanese applications in this review (Table 1). Orphan drug designation and hematologic cancer (Table 3) were identified as factors when using SEPs (Table 3). We believe that for rare cancers, there is no choice but to use SEPs when considering feasibility, and for many hematologic cancers, SEPs are considered valid. However, our study found that priority reviews are less likely to use SEPs. We have speculated that the presence of OS data will make it easier to obtain a priority review in Japan.

In this study, we have examined the current situation in postmarketing confirmatory studies of anticancer drugs in Japan and whether the results of OS and clinical benefits were shown. Although there are some research results overseas, this is the first such study to focus on Japanese data to the best of our knowledge. Of the SEP-approved anticancer drugs, 37 approvals (17.5%) had undergone a confirmatory study with OS as the end point, and 65 approvals (30.7%) had not. Recently approved drugs may still be in the planning stage, but given that this study is a survey of anticancer drugs approved over the past 20 years, the implementation rate is low. In addition, more than one-half of the anticancer drugs (22 approvals [59.5%]) that had undergone confirmatory studies were approved in re-examination, even though efficacy was not recognized in terms of OS. We investigated the reasons for approval, which could not be determined from the re-examination report, and, in many cases, the logical background for the approval was unclear. However, no considerable safety issues were noted. We believe it will be necessary to assess further judgments on the basis of the results of the confirmatory studies in re-examinations. Recently, from the viewpoint of patient access, not only the industry but also regulatory authorities have made efforts in the production of innovative drugs that are expected to be effective and available to patients as soon as possible.^37,38,39^ This is not only true in Japan. The US has systems such as breakthrough therapy designation,^40^ Accelerated Approval, and Priority Review,^41^ and Europe has systems such as PRIME (priority medicines), EU Accelerated Assessment, and EU Conditional Marketing Authorization.^42^ In addition, anticancer drugs are often used to target rare diseases,^43^ and Japan, the US, and Europe are each promoting the clinical development of drugs for rare diseases.^44,45,46^ Similar expedited approval systems are also used in Switzerland, Canada, and Australia for the early approval of anticancer drugs.^47^ Furthermore, since the 2017 reforms in the development of anticancer drugs, measures to promote early approval and development have been strongly promoted in China.^48^ In such early approval efforts, a pivotal study with an SEP is considered inevitable as a clinical trial design. However, at the same time as the aforementioned early approval efforts, it is becoming clear that the FDA has limited data on the clinical benefits of novel cancer drugs at the time of approval^49,50^ and that the number of randomized clinical trials at the time of approval is decreasing.^51^ These 2 points have been identified as issues that need to be resolved. In the US, approximately 60% of the anticancer drugs approved in a single-group pivotal study with response rate as the end point have not been subjected to postmarketing randomized clinical trials.^36^ Many anticancer drugs approved under accelerated approval have not undergone confirmatory studies,^52,53^ and their results have not been published.^54^ In the EU, it has been reported that approximately one-third of anticancer drugs cannot complete the postmarketing requirement 5 years after approval.^55^ There are also reports that OS data are important for reimbursement from the National Institute for Health and Care Excellence.^56,57^ These results are not significantly different from our study results in Japan. However, it is very disappointing that similar results were obtained, as Japan has a postapproval reverification system called re-examination and re-evaluation. The revalidation system is formal, and it can be inferred that judgments regarding the usefulness of drugs after approval are not appropriate.

### Limitations

Our study has several limitations. First, it was based solely on publicly available information, and we could not access internally held information within companies or regulatory agencies. Second, this study only targeted approved drugs; discontinued and disapproved drugs were not included. Third, many confirmatory survival clinical trials included in the study were conducted outside Japan. Therefore, we cannot rule out the possibility that ethnic variations and differences in treatment practices in different countries may have affected OS results.

## Conclusions

In conclusion, we have summarized the current situation for SEPs in Japan from 2001 to 2020. Early approval of anticancer drugs is expected to improve patient access; consequently, SEPs are expected to be used in the future. To that end, it is necessary to consider a mechanism by which regulatory authorities can specify SEPs that can be used in Japan. Promoting early approval through SEPs and ensuring the implementation of postmarketing commitment studies are considered useful. Complete follow-up of the true end points in postmarketing studies is required when using SEPs.

## Notes

Supplement 1. **eTable 1. **Supplemental Information and the Details of the Methods Employed in This Study
eReferences
**eTable 2.** Univariate Logistic Regression Analysis for the Acceptance of Surrogate End Points and Each Background Factor **eFigure.** Confirmatory Studies Using Overall Survival After Approval

Supplement 2.
Data Sharing Statement
