# Supplementary material for: Assessment of Surrogate End Point Trends in Clinical Trials to Approve Oncology Drugs From 2001 to 2020 in Japan
Source: JAMA Netw Open. 2023 Apr 28;6(4):e238875. doi: 10.1001/jamanetworkopen.2023.8875 (PMC10148198; doi:10.1001/jamanetworkopen.2023.8875)
Supplement: Supplement 1. — eTable 1. Supplemental Information and the Details of the Methods Employed in This Study eReferences eTable 2. Univariate Logistic Regression Analysis for the Acceptance of Surrogate End Points and Each Background Factor eFigure. Confirmatory Studies Using Overall Survival After Approval [file jamanetwopen-e238875-s001.pdf]

Supplemental Online Content

Maeda H, Shingai R, Takeda K, Hara A, Murai Y, Ofuchi M. Assessment of surrogate end point trends in clinical trials to approve oncology drugs from 2001 to 2020 in Japan. *JAMA Netw Open*. 2023;6(4):e238875. doi:10.1001/jamanetworkopen.2023.8875

eTable 1. Supplemental Information and the Details of the Methods Employed in This Study
eReferences
eTable 2. Univariate Logistic Regression Analysis for the Acceptance of Surrogate End Points and Each Background Factor
eFigure. Confirmatory Studies Using Overall Survival After Approval

This supplemental material has been provided by the authors to give readers additional information about their work.

© 2023 Maeda H et al. *JAMA Network Open.*

eTable 1. Supplemental Information and the Details of the Methods Employed in This Study

Item	Method details
Ethics	This study did not include individual patient records. Instead, it used publicly available data. Therefore, institutional review board approval and patient informed consent were not necessary.
Selection of drugs and database	In this study, we have investigated the oncology drugs approved in Japan from January 2001 to December 2020. We included initial New Drug Applications (iNDAs) as new molecular entities as well as additional indication supplemental NDAs (sNDAs). Approvals for new formulations of a drug for a comparable indication were not included. We searched for clinical trials in Japan using four databases: Clinicaltrials.gov, Japic Clinical Trials Information (JapicCTI), University Hospital Medical Information Network Clinical Trial Registration System (UMIN-CTR), and The Japan Medical Association Center for Clinical Trials. In addition, ClinicalTrials.gov was used to search for clinical trials conducted overseas, including the United States. We used "phase 3," "overall survival (OS)," "each anticancer drug," and "each indication" as our search terms.
Data collection (approval of drugs)	Data were collected from publicly available databases through the Pharmaceuticals and Medical Devices Agency (PMDA) website (http://www.pmda.go.jp/english/index.html). We used the same methods of data collection and extraction as those employed in our previous study.[1] Molecular-targeted agents are class of drugs having various mechanisms of action. For example, monoclonal antibodies and small molecules, such as tyrosine kinase and tumor angiogenesis inhibitors, were considered molecular-targeted agents in this study. Furthermore, clinical development strategies in Japan were classified into four development categories: global multi-regional strategy relying on joint international studies, bridging strategy utilizing data from overseas clinical studies, domestic strategy utilizing data only from Japanese clinical studies, and others. For endpoint evaluations, all clinical trials in the clinical data package at the time of approval were investigated but only one pivotal trial for each indication was selected from among them. The selected pivotal trial was the largest and most relevant to the indication at the time of approval. Of December 31, 2021 Additionally, when the primary study endpoint was not clearly articulated and multiple endpoints met statistical criteria, the endpoint with the highest priority was used for the analysis, using the following hierarchy: survival (e.g., OS, median survival, 3-year survival), progression free survival (progression-free survival,), time-to-event (time to progression, time to recurrence), disease free survival (disease free survival, metastasis free survival, event free survival), and response rate (e.g., tumor or other surrogate response rate). The response rates included hematologic responses to hematologic cancer and testosterone castration rates for prostate cancer. Other endpoints included symptomatic score, PK, and those without specific clinical trial endpoints, such as publicly known applications. For example, if there was no significant difference in OS and there was a significant difference in progression-free survival (PFS), we considered the drug to be approved based on the PFS.

© 2023 Maeda H et al. *JAMA Network Open.*

Data collection (confirmatory studies)	After approval based on surrogate endpoint (SE), when examining whether a confirmatory study was conducted, we specifically investigated whether a phase 3 or 4 study that included OS as an endpoint was conducted after approval. These studies could be conducted in Japan or other countries. Furthermore, we collected this information based on the data available as of December 31, 2021. The clinical trial details, including those of ongoing trials, were obtained from public databases, such as Clinicaltrial.gov and UMIN-CTR . The confirmatory study status was classified into one of the following categories: completed confirmatory study (a phase 3 study or post-marketing study using OS an endpoint has been completed in Japan or overseas); ongoing confirmatory study (the study has been confirmed to be ongoing); waiver of confirmatory study (no need to conduct a confirmatory study due to the availability of a bridging study or public-knowledge application); not yet conducted confirmatory (study to be conducted in the future as the re-examination period has not yet ended). Information on the US Regulatory measures that utilized the drugs for the US NDA review was collected from the Food and Drug Administration (FDA) website (http://www.accessdata.fda.gov/scripts/cder/drugsatfda/).
Data abstraction	This study was prepared in accordance with Strengthening the Reporting of Observational Studies in Epidemiology (STROBE) reporting guidelines [2] for cross-sectional studies. We extracted characteristics of the drugs, indications, clinical and regulatory details, and features of all eligible clinical trials. Riko Shingai (R.S.), Yuna Mutai (Y.M.), Asuka Hara (A.H.), and Momoka Ofuchi (M.O.) conducted the extractions of regulatory and clinical details for each oncology drug. Any questions about regulatory and clinical characteristics were discussed between all authors and were adjusted when necessary. Any disagreement was resolved by a third reviewer [Hideki Maeda (H.M.) or Kentaro Takeda (K.T.)].
Statistical analysis	Descriptive statistics were used to characterize the samples of the new drugs and their indications. The chi-squared test was used to evaluate the changes in endpoints for pivotal clinical trial-supported approvals. A logistic regression model was used to examine the relationship between the SEs and background factors. A univariable logistic regression model and a multivariable logistic model were used to examine the relationship between the SEs and background factors. A univariable logistic regression model was applied for each background factor. All background factors were used in the multivariable logistic model analysis, and the model with the smallest Akaike information criterion (AIC) was selected among all the possible models. The statistical significance level was set at 0.05 for a two-sided test . All analyses were conducted using the SAS 9.4 and JMP Pro ver15 analytical tools.

eReferences

1: Maeda H, Kurokawa T. Acceptance of surrogate endpoints in clinical trials supporting approval of drugs for cancer treatment by the Japanese regulatory agency. *Ann Oncol*. 2015;26:211-216. doi:10.1093/annonc/mdu500

2: 33) Vandenbroucke JP, von Elm E, Altman DG, et al. Strengthening the reporting of observational studies in epidemiology (STROBE). Explanation and elaboration. *Epidemiology*. 2007;18:805-835.

© 2023 Maeda H et al. *JAMA Network Open.*

eTable 2. Univariate Logistic Regression Analysis for the Acceptance of Surrogate End Points and Each Background Factor

Parameter		Estimate	Standard Error	P value	95% Confidence Intervals	
NDA	Intercept	0.667	0.034	<.001	0.600	0.734
	Parameter	0.104	0.053	0.052	-0.001	0.209
Mode of acition	Intercept	0.508	0.081	<.001	0.349	0.667
	Parameter	0.085	0.032	0.009	0.021	0.149
Companion diagnostics	Intercept	0.681	0.031	<.001	0.620	0.741
	Parameter	0.103	0.059	0.081	-0.013	0.218
Tumor type	Intercept	0.722	0.031	<.001	0.660	0.783
	Parameter	-0.044	0.058	0.453	-0.158	0.071
Solid cancer / hematologic cancer	Intercept	0.945	0.045	<.001	0.857	1.033
	Parameter	-0.339	0.054	<.001	-0.445	-0.233
Limitation of indication	Intercept	0.721	0.032	<.001	0.658	0.783
	Parameter	-0.036	0.057	0.521	-0.148	0.075
Special designation by PMDA - Orphan drug designation	Intercept	0.612	0.032	<.001	0.549	0.675
	Parameter	0.262	0.052	<.001	0.159	0.365
Special designation by PMDA - Normal Application	Intercept	0.697	0.032	<.001	0.633	0.761
	Parameter	0.036	0.056	0.522	-0.074	0.145
Special designation by PMDA - Priority Review	Intercept	0.797	0.026	<.001	0.745	0.849
	Parameter	-0.495	0.063	<.001	-0.618	-0.371
Special designation by PMDA - Public knowledge-based application	Intercept	0.714	0.028	<.001	0.659	0.768
	Parameter	-0.047	0.088	0.592	-0.220	0.125
Special designation by PMDA - Expedited review	Intercept	0.711	0.027	<.001	0.658	0.764
	Parameter	-0.045	0.121	0.712	-0.282	0.193
Special designation by PMDA - Sakigake designation	Intercept	0.703	0.027	<.001	0.651	0.755

© 2023 Maeda H et al. *JAMA Network Open.*

	Parameter	0.297	0.187	0.114	-0.071	0.665
Special designation by PMDA - Pediatric disease designation	Intercept	0.705	0.026	<.001	0.653	0.757
	Parameter	0.295	0.229	0.198	-0.155	0.745
Development style	Intercept	0.783	0.086	<.001	0.613	0.953
	Parameter	-0.029	0.032	0.369	-0.093	0.035
Using foreign clinical data for NDA package in Japan	Intercept	0.830	0.062	<.001	0.708	0.952
	Parameter	-0.147	0.068	0.032	-0.282	-0.012
Special committee on unapproved drugs in Japan	Intercept	0.722	0.029	<.001	0.664	0.780
	Parameter	-0.067	0.067	0.316	-0.198	0.064
All case investigations after approval in Japan	Intercept	0.668	0.033	<.001	0.603	0.734
	Parameter	0.105	0.054	0.051	0.000	0.211
Postmarketing clinical study requirement in Japan	Intercept	0.690	0.027	<.001	0.638	0.743
	Parameter	0.310	0.109	0.005	0.094	0.525
Special designation by FDA - Fast track designation	Intercept	0.712	0.028	<.001	0.657	0.766
	Parameter	-0.024	0.085	0.778	-0.192	0.144
Special designation by FDA - Priority review and/or orphan designation	Intercept	0.702	0.031	<.001	0.641	0.763
	Parameter	0.027	0.059	0.655	-0.090	0.143
Special designation by FDA - Accelerated approval	Intercept	0.691	0.028	<.001	0.636	0.746
	Parameter	0.147	0.080	0.066	-0.010	0.304
Special designation by FDA - Breakthrough therapy designation designation	Intercept	0.701	0.027	<.001	0.649	0.754
	Parameter	0.208	0.139	0.138	-0.067	0.482
FDA approval when Japan approval	Intercept	0.790	0.050	<.001	0.691	0.889
	Parameter	-0.111	0.059	0.060	-0.227	0.005
Number of patients in pivotal clinical study less than 100/ over 100	Intercept	0.740	0.029	<.001	0.682	0.798
	Parameter	0.000	0.000	0.021	0.000	0.000
Domestic company / Foreign company	Intercept	0.787	0.043	<.001	0.701	0.873
	Parameter	-0.122	0.054	0.026	-0.229	-0.015

© 2023 Maeda H et al. *JAMA Network Open.*

Origin of product	Intercept	0.746	0.059	<.001	0.629	0.862
	Parameter	-0.046	0.066	0.490	-0.176	0.084
Biological drug	Intercept	0.768	0.031	<.001	0.707	0.828
	Parameter	-0.200	0.057	0.001	-0.311	-0.088
Formulation	Intercept	0.860	0.084	<.001	0.694	1.026
	Parameter	-0.093	0.050	0.061	-0.191	0.004

© 2023 Maeda H et al. *JAMA Network Open.*

eFigure. Confirmatory Studies Using Overall Survival After Approval

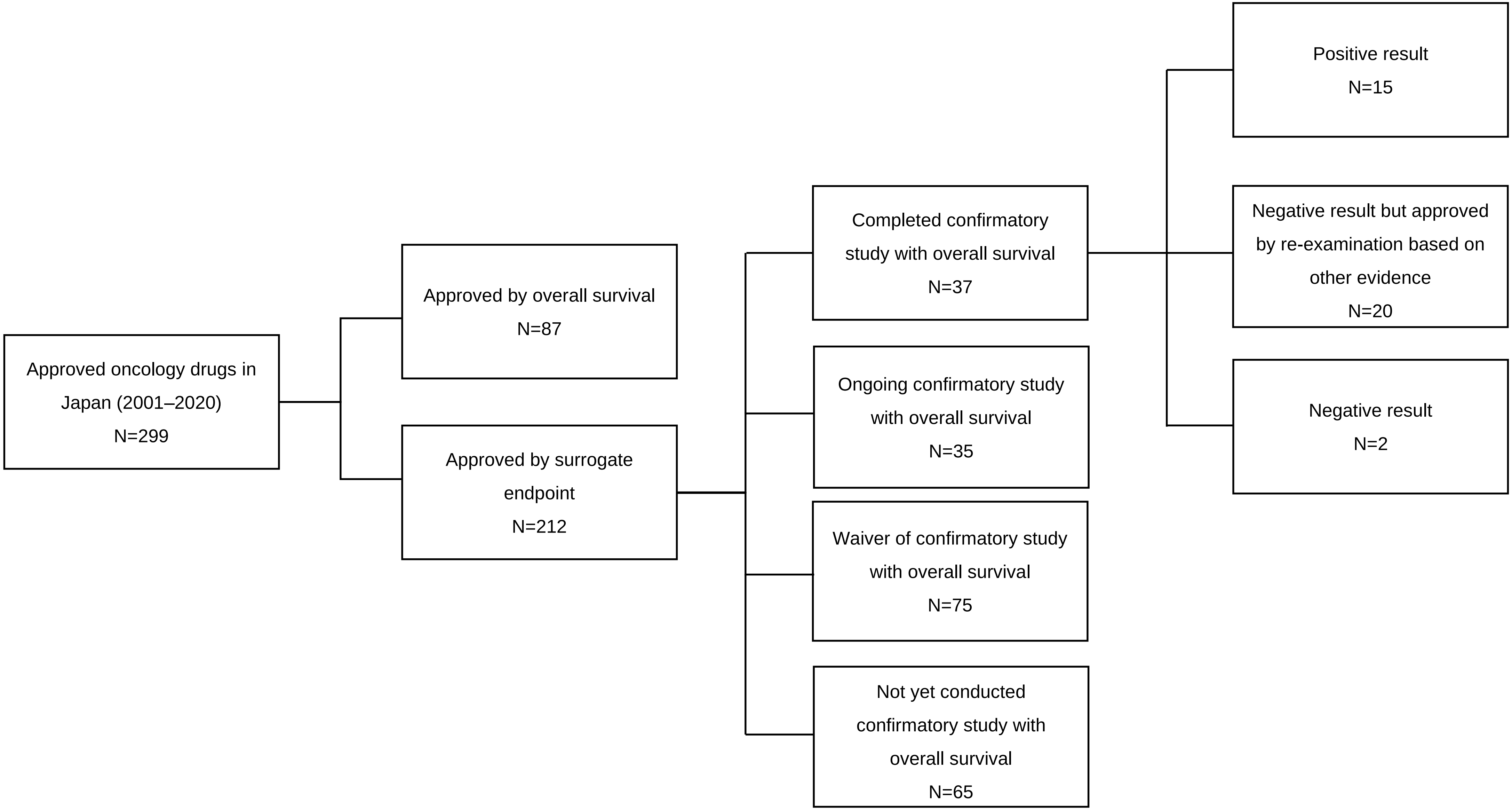

© 2023 Maeda H et al. *JAMA Network Open.*
