# Supplementary material for: Assessment of Surrogate End Point Trends in Clinical Trials to Approve Oncology Drugs From 2001 to 2020 in Japan
Source: JAMA Netw Open. 2023 Apr 28;6(4):e238875. doi: 10.1001/jamanetworkopen.2023.8875 (PMC10148198; doi:10.1001/jamanetworkopen.2023.8875)
Supplement: Supplement 2. — Data Sharing Statement [file jamanetwopen-e238875-s002.pdf]

Data Sharing Statement

Maeda. Assessment of Surrogate End Point Trends in Clinical Trials to Approve Oncology Drugs From 2001 to 2020 in Japan. *JAMA Netw Open*. Published April 28, 2023. doi:10.1001/jamanetworkopen.2023.8875

Data
Data available: Yes
Data types: Data (not involving human participants)
How to access data: Data available on request from the researchers after approval by the author.
When available: With publication

Supporting Documents
Document types: None

Additional Information
Who can access the data: Researchers whose proposed use of the data has been approved
Types of analyses: For any purpose or for a specified purpose
Mechanisms of data availability: After approval of a proposal
